# Imidazole Analogs of Vascular-Disrupting Combretastatin A-4 with Pleiotropic Efficacy against Resistant Colorectal Cancer Models

**DOI:** 10.3390/ijms222313082

**Published:** 2021-12-03

**Authors:** Franziska Reipsch, Bernhard Biersack, Henrike Lucas, Rainer Schobert, Thomas Mueller

**Affiliations:** 1 University Clinic for Internal Medicine IV, Hematology/Oncology, Medical Faculty, Martin Luther University Halle-Wittenberg, 06120 Halle (Saale), Germany; franziska.reipsch@uk-halle.de; 2 Organic Chemistry Laboratory, University of Bayreuth, Universitätsstraße 30, 95440 Bayreuth, Germany; bernhard.biersack@uni-bayreuth.de (B.B.); rainer.schobert@uni-bayreuth.de (R.S.); 3 Institute of Pharmacy, Martin Luther University Halle-Wittenberg, 06120 Halle (Saale), Germany; henrike.lucas@pharmazie.uni-halle.de

**Keywords:** combretastatin A-4, imidazoles, vascular-disrupting agents, microtubule destabilization, chemotherapy resistance, colorectal cancer

## Abstract

Specific targeting of the tumoral vasculature by vascular-disrupting agents (VDA), of which combretastatin A-4 (CA-4) is a main representative, has been considered a new therapeutic strategy against multidrug-resistant tumors. In addition, CA-4 and analogs are tubulin-targeting agents and can exert direct antitumor effects by different mechanisms. Herein, we analyzed a series of synthetic CA-4 analogs featuring *N*-methylimidazole-bridged *Z*-alkenes with different halo- or amino-substituted aryl rings in vitro and in vivo, focusing on models of colorectal cancer. Combined in vitro/in vivo structure–activity relationship studies using cell lines and xenograft tumors susceptible to VDA-induced vascular damage demonstrated a clear association of cytotoxic and vascular-disrupting activity with the ability to inhibit tubulin polymerization, which was determined by specific substitution constellations. The most active compounds were tested in an extended panel of colorectal cancer (CRC) cell lines and showed activity in CA-4-resistant and chemotherapy-resistant cell lines. The bromo derivative brimamin was then compared with the known fosbretabulin (CA-4P) by activity tests on DLD-1- (multidrug-resistant) and HT29- (CA-4-resistant) derived xenograft tumors. Treatment did not induce pronounced vascular-disrupting effects in these tumors. Histological analyses revealed distinct tumor substructures and vessel compositions of DLD-1/HT29 tumors, which clearly differed from the tumor models susceptible to VDA treatment. Even so, brimamin effectively retarded the growth of DLD-1 tumors, overcoming their resistance to standard treatment, and it inhibited the outgrowth of disseminated HT29 tumor cells in an experimental metastasis model. In conclusion, combretastatin analogous *N*-methylimidazoles proved capable of inducing vascular-disrupting effects, comparable to those of CA-4P. In addition, they showed antitumor activities in models of drug-resistant colorectal cancer, independent of vascular-disrupting effects.

## 1. Introduction

Chemotherapy of cancer is generally limited by the high incidence of malignant tumors that display resistance to a range of chemical antitumor agents, either intrinsic or acquired upon repeated administration. Specific targeting of the tumoral vasculature by compounds cumulatively referred to as low molecular weight vascular-disrupting agents (VDA) has been considered a new therapeutic strategy against multidrug-resistant tumors. VDA destroy established tumor vasculature as distinguished from antiangiogenic agents, which prevent tumor neovascularization by inhibiting proangiogenic factors [1]. The naturally occurring compound combretastatin A-4 (CA-4) and its water-soluble prodrug combretastatin A-4 phosphate (CA-4P, fosbrestabulin), which are among the most extensively studied VDA, belong to the large group of tubulin-binding VDA [2,3,4,5]. Tubulin provides an essential structural support to immature tumor vasculature. The selectivity of tubulin-binding VDA for tumor vasculature, sparing normal vasculature, is due to the relative immaturity and instability of aberrant tumor vasculature [2]. CA-4 binds primarily to the colchicine-binding site of beta-tubulin and inhibits microtubule polymerization. This affects the microtubule dynamics and stability and causes disruption of the tumor endothelial cell cytoskeleton and junctions between endothelial cells, eventually resulting in changes in endothelial cell shape, leaky vessels, vessel lumen congestion, cessation of blood flow, ischemia, and ultimately tumor hemorrhagic necrosis [2,3,4,5]. In contrast, mature vessels are protected from disruption by an additional structural support provided by the actin cytoskeleton together with a mature basement membrane and vessel-associated pericytes.

Clinical evaluation of CA-4 and its derivatives has primarily been focused on their pronounced vascular-targeting properties. However, the combretastatins are natural tubulin-targeting agents that can directly target cancer cells and can also exert anticancer effects by different mechanisms [6]. In addition, the cis-stilbene motif of CA-4 provides a useful lead structure, which lends itself ideally to synthetic modifications and drug optimization. Great efforts have been made to synthesize derivatives of CA-4 with various substituent patterns on the A- and B-rings and with modifications of the bridging alkene double bond in order to improve the modes of tubulin interaction, the cytotoxicity, the vascular-disrupting activity, and the in vivo applicability [7,8].

Recently, we developed a series of *N*-methylimidazole-bridged CA-4 derivatives bearing different halo/amino substituents at the A- or B-ring and investigated their antitumoral and antivascular properties both in vitro and in vivo, whereby our in vivo xenograft tumor models clearly showed susceptibility to VDA treatment [9,10,11]. In the current study, we compared selected imidazoles in terms of any potential association of their tubulin polymerization inhibition and cytotoxicity, as well as in terms of their vascular-disrupting activity, cytotoxic activity in an extended panel of heterogeneous colorectal carcinoma cell lines, and antitumor activity in colorectal carcinoma xenografts without occurrence of obvious vascular-disrupting effects.

## 2. Results

### 2.1. Cytotoxic and Vascular-Disrupting Activities of CA-4 Analogous Imidazoles Depend on Specific Substitution Patterns and Correlate with Their Ability to Inhibit Tubulin Polymerization

**Figure 1:**
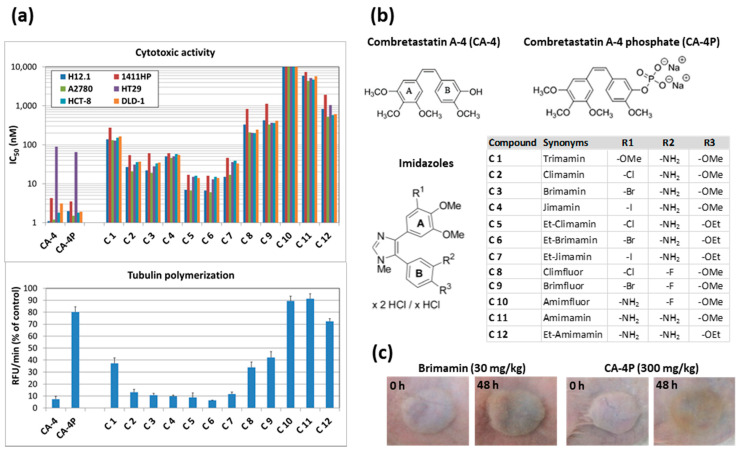
Structure–activity relationship study of imidazoles C 1–C 12 compared with CA-4 and CA-4P. (**a**) Combined cytotoxicity and tubulin polymerization assay. Displayed are the means of IC_50_ values of compounds against a panel of six different cell lines (upper diagram). Tubulin polymerization is quantified as relative fluorescence units per minute and is given as percent of untreated control (lower diagram). (**b**) Structures of combretastatin A-4 (CA-4) and combretastatin A-4 phosphate (fosbretabulin) and of *N*-methyl-4-(4,5-dimethoxyphenyl)-5-phenyl-imidazolium hydrochlorides C 1–C 12 with variation in residues R^1^, R^2^, and R^3^ as specified. (**c**) Representative examples of 1411HP xenograft tumors before and 48 h after treatment, showing typical discoloration indicating a vascular-disrupting effect.

Combined cytotoxicity and tubulin polymerization assays were performed with a series of 12 CA-4 analogous N-methylimidazoles with different halo/amino substitution patterns, including the trimethoxy-substituted compound C 1 [12], in comparison to CA-4 and CA-4P (Figure 1, Table 1). Structures of CA-4/CA-4P and imidazoles are shown in Figure 1b. This structure–activity relationship (SAR) study demonstrated that different substitution constellations can result in quite diverse cytotoxic activities against tumor cells of various entities, ranging from highly cytotoxic with low nanomolar IC_50_ values (for C 5 and C 6) to virtually inactive (e.g., compound C 10 with IC_50_ > 10 µM). The observed cytotoxicities of the tested compounds clearly correlated with their inhibitory effect on the polymerization of purified tubulin (Figure 1a, Table 1). An analysis of the influence of specific substitution patterns on the cytotoxicities and tubulin interference revealed interesting SARs. For instance, the replacement of the methoxy group at R^1^-position on the A-ring of derivative C 1 with halogens led to derivatives (C 2, C 3, and C 4) with enhanced antitubulin and cytotoxic activities. Replacing the methoxy group at R^3^-position on the B-ring of derivatives C 2, C 3, and C 4 with an ethoxy group further improved the activities of the resulting derivatives C 5, C 6, and C 7, with chloro and bromo derivatives outperforming the iodo analogs (Figure 1, Table 1). Keeping the *meta*-bromo or *meta*-chloro substituent at R^1^-position on the A-ring while replacing the *meta*-amino group at R^2^-position on the B-ring with a fluoro group resulted in a decrease in activity (C 2, C 3 vs. C 8, C 9). A loss of inhibitory effects on tubulin polymerization, together with an attenuated or absent cytotoxic activity, was observed when an amino group was placed in R^1^-position on the A-ring, as in derivatives C 10 and C 11. However, additional replacement of the methoxy group in R^3^-position on the B-ring with an ethoxy group led to an increase in activity (C 11 vs. C 12). According to their differential activities, the tested imidazoles could be divided into three groups. Group 1 comprising the most active compounds C 2, C 3, C 4, C 5, C 6, and C 7 inhibited tubulin polymerization by about 90% and showed high cytotoxic activities against cancer cells with two-digit or even one-digit nanomolar IC_50_ values (Figure 1a, Table 1). Compounds in group 2 (C 1, C 8, and C 9) inhibited tubulin polymerization by approx. 60% and still showed cytotoxic activities with lower three-digit nanomolar IC_50_ values against all cell lines apart from the resistant germ cell tumor cell line 1411HP. Within the third group of compounds C 10, C 11, and C 12, the latter had some residual tubulin polymerization inhibitory activity (approx. 30%) and a moderate cytotoxic activity in the upper nanomolar IC_50_ range, whereas C 10 and C 11 had no effect on tubulin dynamics yet differed in their cytotoxic activities. While C 11 showed IC_50_ values in the lower micromolar range, fluoro derivative C 10 was inactive at concentrations below 10 µM (Table 1). Apparently, the different cytotoxicities of C 10 and C 11 are independent of mechanisms associated with tubulin. Together these SAR analyses highlighted the importance of having a chloro or bromo substitution in R^1^-position and an NH_2_ substituent in R^2^-position for efficacy.

CA-4 and CA-4P showed high cytotoxic activity with one-digit nanomolar IC_50_ values and even outperformed the most active imidazoles C 5 and C 6, with the CA-4-resistant cell line HT29 as one exception (Figure 1, Table 1). The typical resistance of the colorectal carcinoma cell line HT29 against CA-4 and analogous compounds bearing an OH-group in R^2^-position on the B-ring was previously reported to be partly mediated by an overexpression of the MRP-1 efflux transporter [13]. Despite similar overall cytotoxic activities of CA-4 and its phosphate prodrug CA-4P, the latter displayed a distinctly lower ability to inhibit the polymerization of purified tubulin. This is likely due to the phosphate group hindering the interaction with neat tubulin under cell-free conditions. In contrast, during the cytotoxicity assay, the prodrug becomes activated by cleavage of the phosphate group after entering the cell.

Next, we tested the vascular-disrupting activity of compounds by applying our 1411HP germ cell tumor nude mouse xenograft model. This is a highly vascularized tumor type, responsive to treatment with vascular-damaging agents that cause typical effects such as intratumoral hemorrhages, visible as a red/blue to brown discoloration of tumors, leading to extensive necrosis [9,10]. All compounds in group 1 induced a typical vascular-disrupting effect upon treatment with a single dose of 30 mg/kg (Figure 1c for examples, Table 1), confirming previous studies with derivatives C 2, C 3, and C 6. Compound C 9 from group 2 was tolerated at higher doses, and treatment with 90 mg/kg was necessary to induce tumor discoloration. Compound C 12 from group 3 could be dosed even higher, and faint signs of discoloration were observed only after administration of 150 mg/kg. Thus, within the series of imidazole compounds, a decreasing ability to inhibit tubulin polymerization was associated with losing overall activity. In the case of CA-4P, a single dose of 300 mg/kg, which was tolerated by mice, was necessary and sufficient to induce a vascular-disrupting effect (Figure 1c). The apparent discrepancy between vastly different in vivo doses of CA-4P and the N-methylimidazoles being necessary to achieve the same vascular-disrupting effect and their similar in vitro activities is likely to originate from their completely different pharmacokinetic profiles.

In addition to the specific CA-4 resistance of HT29 cells, the cisplatin-resistant germ cell tumor cell line 1411HP generally showed a lower sensitivity to CA-4 and the imidazoles when compared with its cisplatin-sensitive counterpart cell line H12.1 but also when compared with the other cell lines (Figure 1a, Table 1). Moreover, 1411HP xenograft tumors are also resistant to treatment with doxorubicin [14]. Nevertheless, treatment with climamin (C 2) or brimamin (C 3) was shown to induce regression and long-term response in drug-resistant 1411HP xenograft tumors, which can be mainly ascribed to the vascular-disrupting activity as the prevalent mechanism of the compounds to overcome resistance in this model [10].

### 2.2. Cytotoxic Activities of CA-4 Analogous Imidazoles in an Extended Panel of CRC Cell Lines with Differential Sensitivity to Conventional Chemotherapeutics

**Figure 2:**
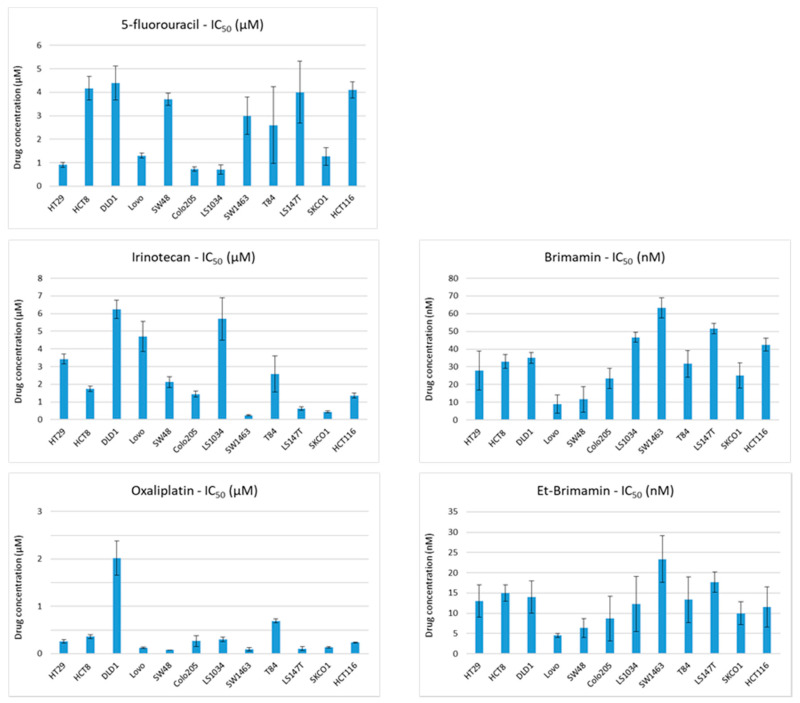
Cytotoxic activities of brimamin and Et-brimamin compared with those of conventional drugs in CRC cell lines. Displayed are the means of IC_50_ values ± standard deviation (*n* = 3).

Similar cytotoxic activities of the imidazoles C 1–C 12 were observed against the three colorectal carcinoma (CRC) cell lines HCT-8, HT29, and DLD-1 (Table 1). Notably, the cell line DLD-1 represents a drug-resistant model that showed a limited response to the clinically relevant combination treatment with 5-fluorouracil (5-FU) and irinotecan in xenograft studies [15]. In contrast, xenografts of HT29 did respond to 5-FU/irinotecan combination treatment [16]. To explore the general activity of our imidazoles against CRC cells, we assessed the cytotoxicity of brimamin (C 3) and Et-brimamin (C 6) in an extended panel of heterogeneous CRC cell lines in comparison to the clinically established chemotherapeutics 5-FU, irinotecan, and oxaliplatin. For this purpose, we performed SRB cytotoxicity assays to generate dose–response curves for the determination of drug-specific IC_50_ values. The cell line- and drug-specific IC_50_ values are depicted in Figure 2. The CRC cell lines clearly differed in their sensitivities to the three chemotherapeutic agents. DLD-1 cells were generally resistant. This is particularly relevant considering that the two typically applied regimens to treat CRC patients are a combination of 5-FU and irinotecan (FOLFIRI), or of 5-FU and oxaliplatin (FOLFOX). Brimamin and Et-brimamin gave a similar cytotoxicity footprint across the CRC cell line panel, with the latter having generally lower IC_50_ values (Figure 2). Compared with the initially tested CRC cell lines HT29, HCT-8, and DLD-1, which showed similar IC_50_ values, the additional nine CRC cell lines exhibited a differential response, with most cell lines being similarly or more sensitive. Notably, the cell lines showing lower sensitivity to brimamin and Et-brimamin were highly sensitive to at least one of the three conventional drugs (Figure 2). Overall, the comparison of all drug response patterns suggested that mechanisms responsible for resistance to the conventional drugs apparently do not affect the response of CRC cells to CA-4 analogous imidazoles in these models.

### 2.3. Lack of Induction of Vascular-Disrupting Effects in CRC Xenograft Tumors Is Associated with Specific Tumor Substructure

**Figure 3:**
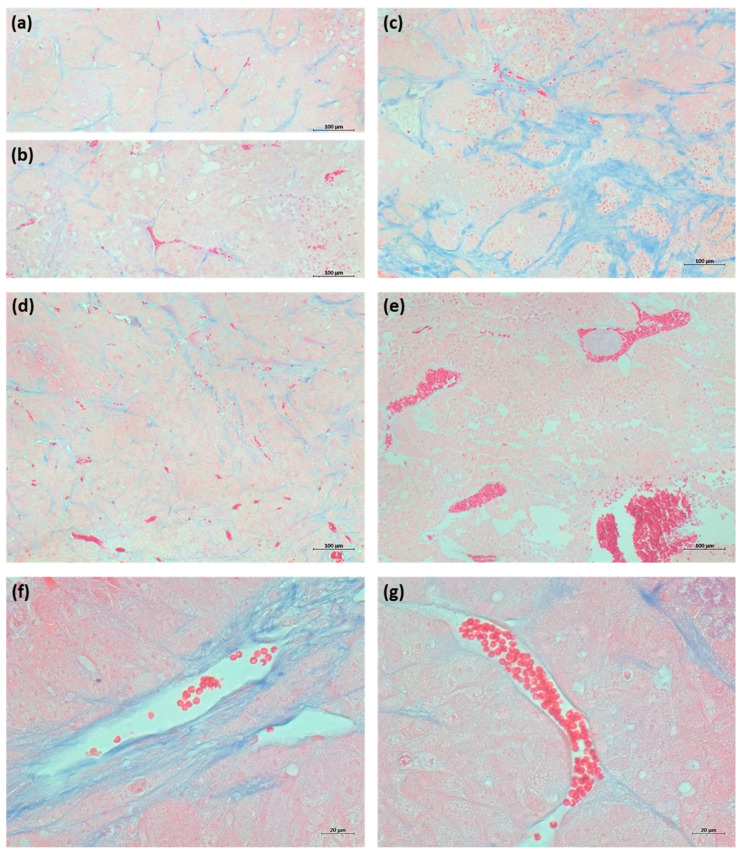
Analysis of tumor substructure after Azan staining. (**a**–**c**) Different areas of HT29 tumors, showing small vessels or capillaries embedded in collagen-rich septal structures or compositions with abundant fibrotic tissue. (**d**) DLD1 tumor showing similar structure to HT29 tumors. (**e**) 1411HP tumor showing large vessels and less extracellular material or stromal structures. (**f**,**g**) Examples of vessels of DLD-1 tumor, representing typical vessel structures found in DLD-1/HT29 tumors, showing distinct vessels with abundant (**f**) or normal (**g**) collagen-based supporting structure. (Scale bars: (**a**–**e**)—100 µm; (**f**,**g**)—20 µm).

Focusing on the CRC model, we chose DLD-1- (multidrug-resistant) and HT29- (CA-4-resistant) derived nude mouse xenograft tumors to investigate any acute tumor response upon a single treatment with brimamin or CA-4P. Surprisingly, the established therapeutic doses of 30 and 300 mg/kg of brimamin and CA-4P, respectively, failed to induce typical vascular-disrupting effects (tumor discoloration) and tumor regression in these models, which is in marked contrast to observations made in the 1411HP tumor model. Histological examinations using HE staining confirmed the absence of treatment-induced intratumoral hemorrhages (not shown). Next, we performed Azan staining to analyze the specific tumor substructure of DLD-1/HT29 vs. 1411HP xenograft tumors. As shown in Figure 3, xenograft tumors of HT29 and DLD-1 were characterized by compacted tumor parenchyma, organized with collagen-rich septal structures containing directly embedded vessels or capillaries. In addition, HT29 tumors occasionally contained areas with abundant fibrotic tissue. Examination of the vasculature in DLD-1/HT29 tumors revealed clearly recognizable vessels with abundant or normal collagen-based supporting structures. In contrast, 1411HP xenografts were highly vascularized and comprised large vessels within loosely organized tumor parenchyma lacking collagen-rich supporting structures (Figure 3). This suggests that the completely different tumor substructure of DLD-1/HT29 tumors, especially the specific vessel composition, might be responsible for the absence of treatment-induced vascular-disrupting effects. The small diameter of vessels together with the adherent, stabilizing stromal structure probably prevents a complete collapse of vessels, which would otherwise lead to the induction of hemorrhagic necrosis.

### 2.4. Antitumor Activity of Brimamin and CA-4P in CRC Xenograft Tumor Models

**Figure 4:**
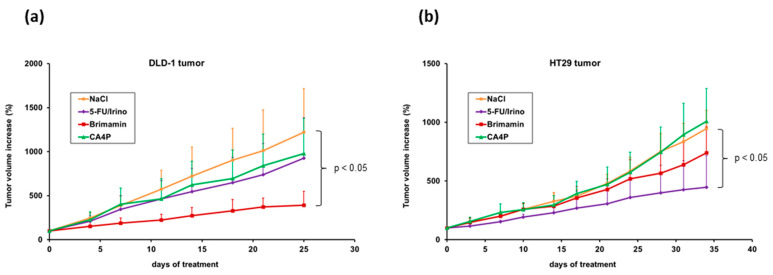
Antitumor activity of brimamin compared with CA-4P and standard treatment. Nude mice bearing s.c. xenograft tumors of DLD-1 (**a**) and HT29 (**b**) were treated with NaCl (control), brimamin (25 mg/kg BW), CA-4P (250 mg/kg BW), or 5-FU + irinotecan (30 mg/kg + 50 mg/kg BW) on days 0, 7, 14, 21, and 28 (only HT29). The tumor volume increase relative to the start of treatment on day 0 is shown, as mean values ± SD (*n* = 6).

Despite the failure of compounds to induce pronounced vascular-disrupting effects in some CRC models, we assessed their overall antitumor efficacy, mediated by vasculature-independent modes of cytotoxic action. Mice bearing HT29 and DLD-1 xenograft tumors were treated with either brimamin, or CA-4P, or the standard FOLFIRI scheme. In the drug-resistant DLD-1 model, brimamin significantly inhibited tumor growth compared with controls (Figure 4a). Standard treatment was less efficient, as expected. CA-4P treatment showed similar limited effects on tumor growth. In the CA-4-resistant HT29 model, standard treatment significantly inhibited tumor growth whereas CA-4P had no effect (Figure 4b). Brimamin exerted activity to some extent but was clearly less efficient compared with the DLD-1 model. These results demonstrate that data obtained from in vitro analyses cannot be simply translated to in vivo tumor models. This is particularly true for CA-4P and brimamin. The particularly high in vitro activity of CA-4P was not reproduced in vivo, although in vitro resistance of HT29 clearly correlated with the lack of response in HT29 tumors. In addition, although brimamin outperformed CA-4P in both DLD-1 and HT29 tumors, the treatment efficacy clearly differed between the two models despite similar in vitro activities. Notably, brimamin was effective in tumors resistant to the standard regimen.

### 2.5. Antimetastatic Activity of Brimamin in the Resistant DLD-1 Xenograft Tumor Model

**Figure 5:**
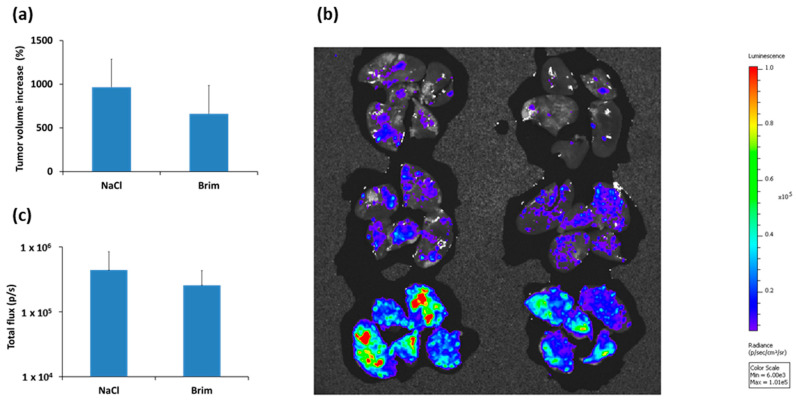
Antimetastatic activity of brimamin in the DLD-1 tumor model. Nude mice bearing s.c. xenograft tumors of luciferase-expressing DLD-1 were treated with NaCl (control) or brimamin (20 mg/kg BW) on days 0, 7, 14, and 21. (**a**) The tumor volume increase on day 25 relative to the start of treatment on day 0 is shown, as mean values ± SD (*n* = 3). (**b**) Ex vivo analysis of lung metastasis using bioluminescence imaging. (**c**) Quantification of metastatic burden given as total flux (photons per seconds).

Next, we tested the antimetastatic activity of brimamin in the DLD-1 tumor model. For this purpose, we used a stably luciferase-expressing variant of DLD-1 to generate subcutaneous tumors, enabling the analysis of lung metastasis. Mice were treated with brimamin according to the scheme above but with a lower dose of 20 mg/kg to achieve a lesser growth inhibition of the primary tumor. After completion of the treatment course, lungs were removed and analyzed ex vivo by bioluminescence imaging. The treatment with brimamin resulted in an attenuated growth retardation compared with treatments at higher doses (Figure 5a). Occurrence of lung metastasis was only minimally reduced (Figure 5b,c).

### 2.6. Antimetastatic Activity of Brimamin in an Experimental Metastasis Model

**Figure 6:**
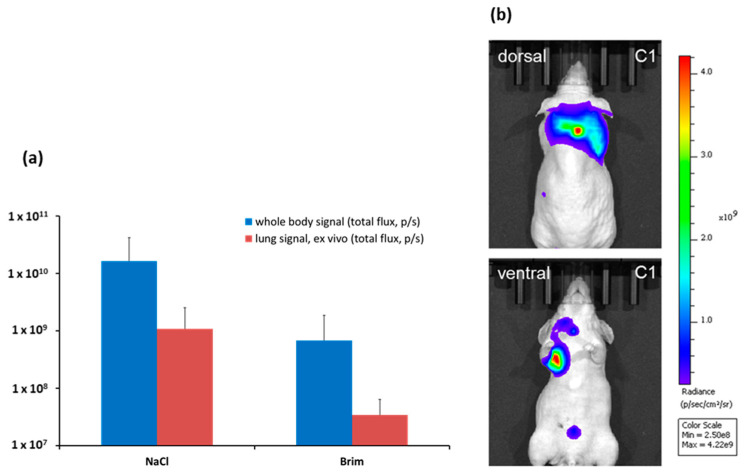
Antimetastatic activity of brimamin in the HT29 tumor model of disseminated tumor cells. Nude mice were injected i.v. with luciferase-expressing HT29-Luc cells, and then treated with NaCl (control) or brimamin (25 mg/kg BW) once weekly. (**a**) The whole tumor burden and lung metastasis are shown (mean values ± SD (*n* = 5)) as analyzed by bioluminescence imaging and are given as total flux (photons per seconds). (**b**) An example from the control group showing tumor growth in the nape, flank, chest, neck, and abdomen.

In a model simulating the setting of adjuvant chemotherapy, luciferase-expressing HT29 cells were injected i.v., which eventually led to a metastatic outgrowth of disseminated tumor cells. Mice were monitored using in vivo bioluminescence imaging. After 5 weeks of treatment, whole body imaging was performed to quantify the overall tumor burden. In addition, lungs were analyzed ex vivo. As shown in Figure 6, the treatment with brimamin reduced the overall tumor burden and led to a decrease in lung metastasis.

## 3. Discussion

In the current study, we analyzed a series of synthetic combretastatin A-4 analogs, featuring *N*-methylimidazole-bridged Z-alkenes with different halo- or amino-substituted aryl rings, for their antitumor effects in vitro and in vivo with a focus on models of colorectal cancer. Naturally occurring compounds with specific bioactivities are often used as a springboard for the development of new therapeutic agents to treat cancer. The cis-stilbene motif of CA-4 is a particularly useful and fruitful lead structure. Scores of synthetic derivatives of CA-4 with various structural modifications to improve, adjust, or balance its biological properties, such as tubulin interaction, cytotoxicity, vasculature-disrupting activity, and in vivo applicability, have been reported [7,8]. Modifications on the A- and B-rings are mainly aimed at enhancing the tubulin affinity, whereas the strategy of bridging the double bond is mainly aimed at stabilizing the cis-stilbene motif to prevent trans-isomerization and loss of activity. Our combined in vitro and in vivo studies on the structure–activity relationships of imidazoles C 1–C 12 confirmed an association between their cytotoxic and antivascular activities and their ability to inhibit tubulin polymerization, which is determined by specific substitution patterns. This finding corroborates previous studies by ourselves and by other groups, and it suggests that the preservation of tubulin affinity is likely also crucial for the overall anticancer activity of new CA-4 derivatives. The most active imidazoles of the series investigated here are comparable to, yet not superior to, the lead CA-4 in terms of tubulin interaction and in vitro cytotoxicity. However, most of the active imidazoles discussed here are water-soluble, rendering costly prodrug strategies unnecessary. They also show efficacy in CA-4-resistant cancer cells, which is mainly mediated by the imidazole ring and the amino group in R^2^-position. Moreover, they clearly differ from CA-4 in their in vivo characteristics. Therapeutically applicable dosages to induce vascular-disrupting and antitumor effects are ten-fold lower for the imidazoles when compared with CA-4P, in contrast to their in vitro similarity. This may be due to their distinctly different pharmacokinetic profiles, including different mechanisms of accumulation and detoxification of CA-4P vs. N-methylimidazoles, resulting in different therapeutic windows.

An interesting observation was the failure of CA-4P as well as brimamin to induce pronounced vascular-disrupting effects in both DLD-1 and HT29 colorectal cancer models, as they had done previously in the 1411HP tumor model. In our previous studies, we used 1411HP tumors and also xenografts of the ovarian cancer cell line A2780cis as models to analyze the ability of compounds to induce vascular-disrupting effects in association with the potential to overcome drug resistance. Both models, and in particular the 1411HP tumors, were susceptible to treatment with VDA. They are highly vascularized, fast-growing tumors comprising large vessels and less stromal-based supporting structures and are prone to hemorrhages. Therefore, the potential to overcome drug resistance in these models can be mainly ascribed to the vascular-disrupting activity of compounds as the prevalent mechanism. Treatment of DLD-1 or HT29 tumors did not induce pronounced vascular-disrupting effects, which can be explained by their completely different tumor substructure, especially the specific vessel composition. Overall, antitumor activity observed in models not susceptible to VDA is mainly the result of direct cytotoxicity against tumor cells and other indirect mechanisms. Indeed, it has been shown that combretastatins and analogous compounds, in addition to being tubulin-targeting agents, can directly target cancer cells and may exert anticancer effects by other mechanisms of action [6].

One important prerequisite to achieve VDA-induced antitumor activity in drug-resistant tumors independent of vascular-disrupting effects is a lack of cross-resistance of the compounds to the relevant conventional chemotherapeutic drugs. In this regard, the efficacy of brimamin in the drug-resistant DLD-1 tumor model is an apt example. The direct cytotoxicity against tumor cells observed in vitro could be reproduced in vivo as an impressive antitumoral effect. However, such a direct translation from in vitro activity to in vivo efficacy is not generally achieved, as seen in the case of CA-4P, which showed restricted efficacy in vivo despite its even higher in vitro activity compared with brimamin. This is due to the complexity of in vivo tumor models, which better reflect clinical conditions. It may depend on the specific physicochemical characteristics of the compounds themselves and their specific interaction with the in vivo system, ranging from pharmacokinetic effects to intratumoral mechanisms mediated by different characteristics of tumor tissues such as the tumor substructure and the tumor microenvironment. Moreover, the complete lack of any antitumor activity by CA-4P in the HT29 tumor model is another example showing the effect when a lack of vascular-disrupting activity is combined with a lack of direct cytotoxicity because of resistance of tumor cells to the compound. This is in accordance with observations made by Lunt et al. [17]. Using tumor cells and xenografts with acquired CA-4P resistance, they showed that vascular damage dominates the therapeutic response to this agent. Brimamin was able to overcome the CA-4P resistance of HT29 cells and therefore showed some in vivo activity against HT29 tumors. However, it exerted only a restricted efficacy when compared with its performance in the DLD-1 model, despite an even slightly higher in vitro activity. Since both HT29 and DLD-1 xenograft tumors showed similar substructures including size and composition of vessels, other tumor tissue-specific characteristics such as the tumor-specific microenvironment may contribute to the diverging response. Nevertheless, brimamin clearly inhibited metastatic outgrowth from disseminated HT29 cells in the model simulating the setting of adjuvant chemotherapy. This confirms its antitumor activity when treatment is targeted at individual tumor cells or avascular micrometastases. The analysis of metastasis in the DLD-1 model, comprising the whole metastatic cascade, revealed only a minor reduction, which might be simply associated with the treatment-related retardation of primary tumor growth.

## 4. Materials and Methods

### 4.1. Cell Culture

The following colorectal cancer cell lines were used: HCT-8 (ATCC-CCL-244), HT29 (ATCC-HTB-38), DLD-1 (ATCC-CCL-221), LOVO (ATCC-CCL-229), SW48 (ATCC-CCL-231), LS1034 (ATCC-CRL-2158), SW1463 (ATCC-CCL-234), T84 (ATCC-CCL-248), COLO205 (ATCC-CCL-222), LS174T (ATCC-CL-188), SKCO-1 (ATCC-HTB-39), and HCT116 (ATCC-CCL-247). The ovarian cancer cell line A2780 was provided by Prof. G. Bendas (Pharmaceutical Institute, Rheinische Friedrich Wilhelms Universität Bonn, Bonn, Germany). The germ cell tumor cell line 1411HP [18] was provided by Prof. P. W. Andrews (Centre for Stem Cell Biology, University of Sheffield, Sheffield, UK). The germ cell tumor cell line H12.1 [19] was established by the previous group of Prof. H. J. Schmoll (University Hospital Halle, Halle (Saale), Germany) and belongs to our lab.

All cell lines were cultivated with RPMI medium (Sigma-Aldrich, Taufkirchen, Germany) containing 10% fetal bovine serum (BioWest, Nuaillé, France) and 1% penicillin/streptomycin (Sigma-Aldrich) at 37 °C/5% CO_2_ in a humid atmosphere.

### 4.2. Compounds

Imidazoles were synthesized according to protocols described in previous studies [9,10]. Combretastatin A-4 and combretastatin A-4 phosphate (fosbretabulin) were purchased from Absource Diagnostic GmbH (Munich, Germany). Compounds were dissolved in saline, except CA-4 (DMSO).

### 4.3. Cytotoxicity Assay

Cytotoxic activities were analyzed using the SRB cytotoxicity assay to generate dose–response curves for determination of IC_50_ values. Cells were seeded in 96-well plates and were treated with different concentrations of compounds (10,000 to 1 nM) for 96 h. All subsequent steps were performed according to the previously described SRB assay protocol [14].

### 4.4. Tubulin Polymerization Assay

Analyses of tubulin polymerization were performed using the Tubulin Polymerization Assay Kit (Cytoskeleton, Denver, CO, USA) according to the manufacturer’s instructions and as previously described [10].

### 4.5. Animal Studies

Subcutaneous xenograft tumors were generated by inoculation of 5 million tumor cells into the right flank of male athymic nude mice (local breeding, ZMG of Medical Faculty of MLU Halle). Monitoring of tumor growth was performed by caliper measurement and volume calculation using the formula a^2^ × b × π/6, with “a” being the short and “b” the long dimension. Treatments were performed by intraperitoneal injections. Mouse weight and behavior were controlled daily during the course of treatment.

For the assessment of compound-induced vascular-disrupting effects, xenografts of the cell line 1411 HP were generated and used when they reached a volume of about 1 cm^3^. In the case of the imidazoles, treatment started with 30 mg/kg body weight, followed by a three-day observation period to analyze the tumor response (discoloration, growth retardation) and tolerability. The treatment was continued by a stepwise increase (30 mg/kg) of dosage until the occurrence of effects. Fosbretabulin (CA-4P) was administered in steps of 100 mg/kg.

To analyze antitumor activity in colorectal cancer models, HT29- and DLD-1 cells were inoculated. After establishment of tumors, the mice were divided into four groups (*n* = 6) with similar mean tumor volumes and equal volume distribution at the start of treatment. Treatments comprised weekly applications of brimamin (25 mg/kg BW), fosbretabulin (250 mg/kg BW), 5-FU plus irinotecan (30 mg/kg + 50 mg/kg BW), or normal saline (control). The impact of treatments was calculated as tumor volume increase relative to the start of treatment on day 0 (mean values ± SD).

The analysis of antimetastatic effects was accomplished using models comprising stably luciferase-expressing variants of DLD-1 and HT29. Subcutaneous xenograft tumors of DLD-1-Luc cells were generated, treated (20 mg/kg brimamin vs. control, *n* = 4), and monitored as described above. After completion of the treatment course, lungs were removed and incubated in D-luciferine solution for 10 min, and bioluminescence imaging was performed on an IVIS Spectrum (PerkinElmer, Rodgau, Germany). Metastatic burden was quantified using the Living Image^®^ software (PerkinElmer) and given as total flux (photons per seconds). To analyze the impact on metastatic outgrowth from disseminated tumor cells, 10^6^ HT29-Luc cells were injected i.v., and treatment (25 mg/kg brimamin vs. control, *n* = 5) started 3 days after. Mice were monitored using in vivo bioluminescence imaging. After 5 weeks, whole body imaging was performed to quantify the overall tumor burden followed by necropsy. In addition, lungs were removed and analyzed ex vivo using bioluminescence imaging.

### 4.6. Histological Analyses

The xenograft tumors were removed, formalin-fixed, embedded in paraffin, and sliced to perform histological analysis using Azan and hematoxylin/eosin (HE) staining according to standard protocols as described previously [20].

### 4.7. Statistical Analyses

Statistical analyses were performed with IBM SPSS 25 (IBM Cooperation, Singapore). Groups were treated as normally distributed since explorative data analysis as well as Kolmogorov–Smirnov and Shapiro–Wilk tests did not reject a normal distribution. The tumor volume increase at the end of the study relative to the start of treatment on day 0 was set as the dependent variable. The treatment was the influencing factor. Significant differences between treatments were analyzed using the Levene Test (variance homogeneity) prior to one-way ANOVA (group differences) and post hoc Tukey test (identification of significant group differences).

## 5. Conclusions

Herein, we analyzed a series of synthetic CA-4 analogs featuring *N*-methylimidazole-bridged *Z*-alkenes with different halo- or amino-substituted aryl rings in vitro and in vivo. There was a clear association between their cytotoxic and antivascular activities and their ability to inhibit tubulin polymerization, which was determined by specific substitution patterns. The most active imidazoles of the series were comparable to, yet not superior to, the lead CA-4 in terms of tubulin interaction and in vitro cytotoxicity. In in vivo xenograft tumor studies, they were able to induce vascular-disrupting effects comparable in strength to those of CA-4P. Unlike the lead CA-4, they showed antitumor activity in drug-resistant models of colorectal cancer not susceptible to VDA. The specific activity of compounds in resistant CRC cells and tumors warrants further investigation.

## Figures and Tables

**Table 1:** Characteristics of compounds: specific substituents, IC_50_ (96 h) values of cytotoxicity assays, values of tubulin polymerization assays, and doses to induce vascular-disrupting effects.

Compound	Synonyms	R1	R2	R3	Cytotoxic Activity in Cell Lines IC_50_ (nM), Mean (*n* = 3), SD ≤ 15%						Tubulin Polymerization (% of Control) Mean ± SD	Vascular-Disrupting Effect Dose (mg/kg)
					**H12.1 ^a^**	**1411HP ^b^**	**A2780 ^c^**	**HT29 ^d^**	**HCT-8 ^e^**	**DLD-1 ^f^**		
C 1	Trimamin	-OMe	-NH_2_	-OMe	138	276	131	128	152	164	37.3 (4.5)	n.a. ^g^
C 2	Climamin	-Cl	-NH_2_	-OMe	27	54	21	31	36	37	13.2 (2.5)	30
C 3	Brimamin	-Br	-NH_2_	-OMe	22	61	19	28	33	35	10.8 (1.4)	30
C 4	Jimamin	-I	-NH_2_	-OMe	51	61	46	51	58	55	9.8 (1.0)	30
C 5	Et-Climamin	-Cl	-NH_2_	-OEt	7.0	17	6.7	15	16	14	8.9 (3.8)	30
C 6	Et-Brimamin	-Br	-NH_2_	-OEt	6.7	16	6.0	13	15	14	6.4 (0.3)	30
C 7	Et-Jimamin	-I	-NH_2_	-OEt	15	46	17	36	39	33	11.7 (1.6)	n.a.
C 8	Climfluor	-Cl	-F	-OMe	330	831	208	203	198	244	33.9 (4.5)	n.a.
C 9	Brimfluor	-Br	-F	-OMe	428	1138	331	368	361	415	42.2 (4.8)	90
C 10	Amimfluor	-NH_2_	-F	-OMe	>10,000	>10,000	>10,000	>10,000	>10,000	>10,000	89.5 (4.0)	n.a.
C 11	Amimamin	-NH_2_	-NH_2_	-OMe	5870	7433	4367	5197	4697	5760	91.3 (4.1)	n.a.
C 12	Et-Amimamin	-NH_2_	-NH_2_	-OEt	835	1918	524	1042	575	614	72.4 (2.2)	150
CA-4	Combretastatin A-4				1.1	4.3	1.2	90	1.8	3.1	7.3 (2.6)	n.a.
CA-4P	Fosbretabulin				2.0	3.5	1.5	65	1.8	1.9	80.2 (4.4)	300

^a,b^ germ cell tumor cell lines, ^c^ ovarian carcinoma cell line, ^d–f^ colorectal carcinoma cell lines, ^g^ not analyzed.

## Data Availability

The datasets used and analyzed in the current study are available from the corresponding author upon reasonable request.
